# Cost analysis of patients undergoing allogeneic stem cell transplantation or chimeric antigen receptor T-cell therapy in relapsed or refractory diffuse large B-cell lymphoma from a German healthcare payer perspective

**DOI:** 10.1038/s41409-024-02228-z

**Published:** 2024-02-06

**Authors:** P. Ahmadi, S. Ghandili, F. Jakobs, C. Konnopka, A. Morgner-Miehlke, N. Kröger, F. Ayuk

**Affiliations:** 1 Controlling University Medical Center Hamburg, Hamburg, Germany; 2 Department of Stem Cell Transplantation, University Medical Center Hamburg-Eppendorf, Hamburg, Germany; 3 Department of Oncology, Hematology and Bone Marrow Transplantation with Section Pneumology, University Cancer Center Hamburg, University Medical Center Hamburg-Eppendorf, Hamburg, Germany; 4 Department of Hematology and Stem Cell Transplantation, University Hospital Essen, Essen, Germany; 5 Department of Health Economics and Health Services Research, University Medical Center Hamburg-Eppendorf, Hamburg, Germany; 6 Center for Oncology, University Medical Center Hamburg, Hamburg, Germany

**Keywords:** Public health, B-cell lymphoma

CD19-directed chimeric antigen receptor (CAR)-T-cell therapies have improved response and survival outcome and are now considered standard of care [1–3] in diffuse large B-cell lymphoma (DLBCL) but are associated with high upfront costs. To better understand the cost composition of cellular therapy approaches, we comprehensively analyzed the reimbursement and diagnosis structure of relapse or refractory (r/r) DLBCL inpatients who underwent allogeneic hematopoietic stem cell transplantation (allo-HSCT) or CAR-T-cell therapy from a German healthcare payer perspective. All patients included in this analysis were treated between January 2016 and December 2022 at the Department of Stem Cell Transplantation of the University Medical Center Hamburg-Eppendorf (UKE). Administrative data were collected in the form of discharge type, case type, length of hospital stay (LOS, defined as inpatient stay of medical procedure), ICD codes, OPS codes, G-DRG codes (German Diagnosis-Related Group), G-DRG case mix, intensive care unit (ICU) admission, invasive ventilation, date of inpatient admission, date of discharge and case type. The G-DRG reimbursement system classifies inpatient treatments according to the underlying disease and resource consumption and assigns a specific DRG code to each patient based on their diagnosis (ICD codes), treatment procedures (OPS codes), and further individual factors such as age and LOS. To assess more information about the patient population, we used a comorbidity-driven approach using the Charlson Comorbidity Index (CCI), which considers the presence and severity of comorbidities in patients by categorizing diagnosis codes found in administrative data [4]. The DRG system is a classification system that groups patients with similar clinical and resource utilization characteristics into categories. These categories, known as DRGs, serve as the basis for reimbursement within the German healthcare system. The system takes into account various factors, including the patient’s diagnosis, procedures performed, comorbidities, and complications. In this analysis, the CCI was then used, to investigate any association between LOS, ICU admission rate, or DRG costs. Patients with r/r DLBCL treated with allo-HSCT or CAR-T were allocated to Group A and B, respectively. Total inpatient reimbursement data were determined using codes from the DRG, the new diagnostic and treatment methods regulation (NUB), and additional charges “Zusatzentgelte” (ZE). We aimed to identify differences in DRG flat rates as well as total inpatient costs between Group A and B.

Reimbursement data and the CCI of 16 allo-HSCT and 24 CAR-T inpatient cases were analyzed. The median length of stay was 30 days and 37 days for Group A and Group B, respectively. The median DRG flat rate was €52 626 (€44 784 - €57 713) for Group A and €19 913 (€8 028 to €359 664) for Group B. The median total costs (including additional fees and new diagnostic and treatment methods) for Group A were €83 872 (€63 801 - €114 433) and for Group B €335 137 (€283 923 - €705 539). Median values, ranges, and percentages of reimbursement-relevant key figures for Group A and B are presented in Table 1.

**Table 1:** Reimbursement Relevant key-figures.

	A: allo-HSCT (*n* = 16)	B: CAR-T (*n* = 24)	Total (*n* = 40)
**Length of Stay, in days (range)**			
Across all DRGs	30 (24–58)	37 (12–200)	32 (12–200)
**Case-mix index (range)**			
Across all G-DRGs	15.282 (11.693–17.598)	4.230 (2.131–98.349)	14.233 (2.131–98.349)
**ICU admission,** ***n*** **(%)**			
Across all G-DRGs	1 (6.3)	15 (62.5)	16 (40)
**Tocilicumab,** ***n*** **(%)**			
Across all G-DRGs	0 (0)	19 (79%)	
**Cases per G-DRG,** ***n*** **(%)**			
Across all G-DRGs	16 (100)	24 (100)	40 (100)
A04D	3 (18.8)	0 (0)	3 (7.5)
A04E	13 (81.3)	0 (0)	13 (32.5)
A09A	0 (0)	2 (8.3)	2 (5)
A11B	0 (0)	1 (4.2)	1 (2.5)
A13C	0 (0)	1 (4.2)	1 (2.5)
A15C	0 (0)	2 (8.3)	2 (5)
A36B	0 (0)	1 (4.2)	1 (2.5)
R03Z	0 (0)	1 (4.2)	1 (2.5)
R61A	0 (0)	6 (25)	6 (15)
R61B	0 (0)	3 (12.5)	3 (7.5)
R61E	0 (0)	4 (16.7)	4 (10)
R61H	0 (0)	3 (12.5)	3 (7.5)
DRG flat rates*, in € (range)			
Across all G-DRGs	52 626 (44 784–57 713)	19 913 (8 028–359 664)	49 032 (8 028–359 664)
**Additional costs, in € (range)**			
Across all G-DRGs			
NUB	0 (0–1 396)	297 000 (265 000–360 000)	291 218 (0–360 000)
ZE	31 576 (11 440–61 807)	11 617 (416–95 021)	19 867 (416–95 021)
Total costs, in € (range)**			
Across all G-DRGs	83 872 (63 801–114 433)	335 137 (283 923–705 539)	311 752 (63 801–705 539)
**Comorbidities**			
Any malignancy	16 (100)	24 (100)	40 (100)
Metastatic solid tumor	1 (6.3)	7 (29.2)	8 (20)
Diabetes without chronic complication	3 (18.8)	3 (12.5)	6 (15)
Congestive heart failure	2 (12.5)	1 (4.2)	3 (7.5)
Peripheral vascular disease	0 (0)	2 (8.3)	2 (5)
Chronic pulmonary disease	0 (0)	2 (8.3)	2 (5)
Hemiplegia or paraplegia	1 (6.3)	1 (4.2)	2 (5)
Cerebrovascular disease	0 (0)	1 (4.2)	1 (2.5)
Peptic ulcer disease	0 (0)	1 (4.2)	1 (2.5)
Mild liver disease	1 (6.3)	0 (0)	1 (2.5)
Renal disease	0 (0)	1 (4.2)	1 (2.5)
Myocardial infarction	0 (0)	0 (0)	0 (0)
Dementia	0 (0)	0 (0)	0 (0)
Rheumatic disease	0 (0)	0 (0)	0 (0)
Diabetes with chronic complications	0 (0)	0 (0)	0 (0)
Moderate or severe liver disease	0 (0)	0 (0)	0 (0)
AIDS/HIV	0 (0)	0 (0)	0 (0)
**Charlson Comorbidity Index (CCI) (median)**	2	3	

*CAR* chimeric antigen receptor T cell therapy, *allo-HSCT* allogeneic hematopoietic stem cell transplantation, *ICU* intensive care unit, *DRG* diagnosis realted groups, *NUB* Neue Untersuchungs- und Behandlungsmethoden, *ZE* Zusatzentgelte.

*DRG flat rate: Case mix multiplied with base rate of year, **Total Costs: DRG flat rate and additional costs (NUB/ZE).

Definitions of the G-DRG codes: A04D, Bone marrow transplantation / stem cell transfusion, allogeneic, with graft-versus-host disease grade III and IV or except for plasmacytoma, HLA-mismatched, or with complex treatment for multidrug-resistant pathogens; A04E, Bone marrow transplantation / stem cell transfusion, allogeneic, except for plasmacytoma; A09A, Ventilation > 499 h or > 249 h with intensive care complex treatment > 2352 / 1932 / 2208 p., with highly complex surgery or complex OR procedure, age < 16 years, with intensive care complex treatment > 1764 / 1932 / - points or with very complex surgery and intensive care complex treatment > - / 2208 / - points; A11B, Ventilation > 249 h or > 95 h with intensive care complex treatment > 1764 / 1656 / 1656 expense points, with highly complex intervention or best intervention and best intensive care complex treatment > 1764 / 1656 / 1656 expense points Intensive care complex treatment or age < 2 years in case of congenital malformation; A13C, Ventilation > 95 h with complex OR procedure, age < 6 years or with specific OR process and complicated constellation or with intensive care complex treatment > - / - / 1104 points or age < 16 years; A15C, Bone marrow transplantation / stem cell transfusion, autogenous, except for plasmacytoma, age > 17 years, without definite collection or for plasmacytoma, with definite collection or intensive care complex treatment > 392 / 368 / 368 expenditure points; A36B, Intensive care complex treatment > 588 / 552 / 828 and < 981 / 1105 / 1657 expense points for specific diseases and disorders or complicating constellation for failure and rejection of a hematopoietic cell transplant; R03Z, Lymphoma and leukemia with specific OR procedure, with extremely severe CC, or with specific OR procedure with severe CC, or with other OR procedures with extremely severe CC, age < 16 years; R61A, Lymphoma and non-acute leukemia with sepsis or certain complicating constellation or with agranulocytosis, intracranial metastasis, or port implantation, with extremely severe CC, age > 15 years, with highly complicated chemotherapy, or most severe CC; R61B, Lymphoma and non-acute leukemia with sepsis or other complicating constellation or with complicating diagnosis or port implantation, with extremely severe CC, age > 15 years or with extremely severe CC or tumor lysis syndrome, with complicating diagnosis of leukemia or with most severe CC; R61E, Lymphoma and non-acute leukemia without sepsis, without complicating constellation, with agranulocytosis or port implantation or complicating with isolation pathogens or complex diagnosis of leukemia, without extremely severe CC, age > 17 years, without intensive chemotherapy; R61H, Lymphoma and non-acute leukemia without certain complicating factors, without extremely severe CC, without complex diagnosis, without complicating procedure, age > 15 years.

By investigating the short-term costs, reimbursement-relevant key figures, and possible associations between the CCI for both treatment modalities, we were able to demonstrate that patients with CAR-T infusion have higher median total costs during inpatient stay than allo-HSCT patients. For allo-HSCT patients, we determined median total costs of € 83 872, which is in line with the common literature of ranging from € 61,337–€ 133,280, reported by Jakobs et al. [5]. For CAR-T patients, we determined median total costs of € 335 137. However, the bulk of the cost was driven by the cost of the CAR-T cell product, since the CAR-T product accounted for more than 88% (median of € 297 000) of the total cost of inpatient stay rather than resource consumption such as inpatient care, monitoring, or ICU admission represented by DRG flat rates. Our results are in line with Jakobs et al. who reported median treatment costs between € 310 496 and € 340 458, and Huguet and colleagues from France who reported mean costs per hospital stay of € 342 903 for tisagenlecleucel and € 366 562 for axicabtagene ciloleucel, each accounting for more than 80% of the total cost [5, 6]. Noteworthy, if only DRG flat rates are considered, the median value of allo-HSCT was higher (CAR-T: €19 913 vs. allo-HSCT: €52 626) in our cohort. An increase of one point in the CCI resulted in a 23% increase in costs for both groups.

Regarding LOS, both groups had similar LOS (Group A: 30 days, Group B: 37 days). For Group B, we determined a median LOS of 37 days, which deviates from common literature ranging from 15 to 29.5 days LOS [6–9]. Maziarz et al. and Huguet et al. reported LOS for different CAR-T products [6, 10]. We also found different median LOS of 38.5, 25 and 21 for the CAR-T products axicabtagene ciloleucel, tisagenlecleucel, and lisocabtagene maraleucel respectively, and found axicabtagene ciloleucel patients with the highest LOS in line with the literature. Lisocabtagene maraleucel was administered only in 2022, since its EMA authorization in 2022 for Europe. For Group A, we determined a median LOS of 30 days, which is in line with the common literature ranging from 23–51 days, reported by Jakobs et al. or Godora and colleagues [5, 11]. The LOS for the CAR-T group has decreased over the years due to the experience curve (median stay 2019: 69.5 days, to median stay 2022: 23.5 days).

Furthermore, we observed for the first time a correlation between CCI and LOS in the context of CAR-T cell therapy or allo-HSCT in r/r DLBCL. The results of this study are consistent with previous reports that underlined the use of the CCI as a predictor of inpatient length of stay in cancer patients. In the underlying study, a one-point increase in CCI was associated with a 15% increase in LOS for both groups. Including CCI and evaluating its association for DRG costs and LOS is a novelty in this context and has never been evaluated for CAR-T cell-infused patients before. The CCI is a widely used tool to assess the burden of comorbidities in patients. However, it is important to note that the CCI has some limitations. One of them is that it relies on the International Classification of Diseases (ICD) codes to identify comorbidities, which may not always accurately reflect the complexity of the patients’ conditions. In principle, CCI is utilized to predict mortality rates, but it includes comorbidities rarely present in allo-HSCT recipients and has limitations in capturing comorbidities frequently present in these patients [12]. It is important to recognize that our analysis only captures the short-term costs of these therapies. Local practice including ICU referral of CAR-T patients has evolved over time. Initial practice stipulated ICU referral of all patients with ICANS 2 or higher, explaining the relatively high ICU referral rate of patients, who in majority received axi-cel.

We showed that short-term inpatient costs of CAR-T patients are driven largely by the cost of the CAR-T product rather than by inpatient costs illustrated by DRG flat rates. For the first time, we found an association between CCI, DRG flat rates, and hospital LOS in the setting of r/r DLBCL and CAR-T therapy or allo-HSCT. Any increase in CCI leads to an increase in LOS and costs. A one-point increase in CCI was associated with a 15% increase in the hospital length of stay (*p*-value < 0.01) and a 23% increase in costs (*p*-value < 0.01). Other comorbidity indices like Elixhäuser or HCT-CI should be investigated in further studies. Continuous improvement in the understanding and application of CAR-T therapy positively influences the learning curve, translating into improved management and shorter hospital stays for patients undergoing this innovative treatment.
